# qPCR detection of *Mycobacterium leprae* DNA in urine samples of leprosy patients using the *Rlep* gene target

**DOI:** 10.3389/fmolb.2024.1435679

**Published:** 2024-08-13

**Authors:** D. Diana, M. C. Harish

**Affiliations:** ^1^ Molecular Biology and Immunology Division, Schieffelin Institute of Health – Research and Leprosy Centre, Vellore, India; ^2^ Department of Biotechnology, Thiruvalluvar University, Vellore, India

**Keywords:** *Mycobacterium leprae*, non-invasive, urine, DNA extraction, *Rlep* gene target, quantitative PCR

## Abstract

**Background:**

Leprosy, a chronic infectious disease caused by *Mycobacterium leprae*, continues to pose a public health challenge in many parts of the world. Early and accurate diagnosis is crucial for effective treatment and prevention of disabilities associated with the disease. Molecular techniques such as PCR have demonstrated great potential as a diagnostic tool for directly detecting *M. leprae* DNA in different clinical samples, providing better sensitivity and specificity than conventional diagnostic techniques. The objective of this study was to measure the amount of *M. leprae* DNA in leprosy patients’ urine samples using the *Rlep* gene target through qPCR.

**Methods:**

Different clinical samples such as smear, blood, and urine samples were collected from leprosy patients and healthy individuals. Leprosy patients were classified by the Ridley–Jopling classification. The Ziehl–Neelsen staining method was used for the slit skin smear (SSS) samples, and the bacteriological index (BI) was calculated for leprosy patients. DNA extraction and qPCR were performed for all three types of clinical samples using the *Rlep* gene target.

**Results:**

The *Mycobacterial leprae* DNA was successfully detected and quantified in all clinical samples across all types of leprosy among all the study groups using the *Rlep* gene (129 bp) target. The *Rlep* gene target was able to detect the presence of *M. leprae* DNA in 100% of urine, 96.1% of blood, and 92.2% of SSS samples of leprosy patients. Urine samples showed significant differences (p < 0.001) between the control and the different clinical forms and between borderline tuberculoid (BT) and pure neuritic leprosy (PNL) cases. There are significant differences in cycle threshold (Ct) values between control cases and clinical categories (p < 0.001), as well as specific differences within clinical categories (p < 0.001), reflecting the variability in bacterial load and detection sensitivity across different sample types and clinical manifestations of leprosy.

**Conclusion:**

Overall, this study's findings suggest that the qPCR technique can be used to detect *M. leprae* DNA in urine samples of leprosy patients using the *Rlep* gene target. It can also be used for diagnosing the disease and monitoring the effectiveness of anti-leprosy drugs, including multi-drug therapy (MDT), across various leprosy disease groups.

## Introduction

Leprosy, also known as Hansen’s disease, is a neglected chronic infectious disease caused by *M. leprae* (*M. leprae*) and the recently identified *Mycobacterium lepromatosis*. It mainly affects the skin and peripheral nerves. Despite significant progress in diagnosis, treatment, and control efforts, leprosy remains a public health concern in several regions globally, particularly in developing regions with limited access to healthcare and sanitation. According to WHO, approximately 200,000 new cases of leprosy are reported annually (Han et al., 2008; Maymone et al., 2020a; Maymone et al., 2020b).

The disease is primarily prevalent in tropical and subtropical regions, with a significant number of cases in India, Brazil, and Indonesia (Makhakhe, 2021). Even though leprosy is curable with multi-drug therapy (MDT), early detection and treatment of leprosy are crucial for preventing permanent disabilities, reducing transmission, and improving patient outcomes within communities (Smith et al., 2014; Reibel et al., 2015).

Diagnosing leprosy using conventional methods such as clinical examination, slit skin smear microscopy, and histopathology has limitations in sensitivity and specificity, especially in diagnosing early cases where the bacillary load is low or absent (Banerjee et al., 2011; Lockwood et al., 2012; Azevedo et al., 2017; Ghunawat et al., 2018; Alemu Belachew and Naafs, 2019; Quilter et al., 2020).

In recent years, advancements in molecular techniques, specifically PCR-based assays, have revolutionized the field of infectious disease diagnosis by providing better sensitivity, specificity, and rapidity than traditional methods. In leprosy, PCR-based assays, including conventional PCR, nested PCR, digital droplex PCR, multiplex, and quantitative PCR (qPCR), enable the amplification and detection of specific DNA sequences of *M. leprae*, even in samples with low bacterial load. Conventional PCR can detect *M. leprae* DNA in different clinical samples using different gene targets (Turankar et al., 2015; Girma et al., 2018; Cheng et al., 2019; Manta et al., 2022; David et al., 2024). The combination of multi-target, nested PCR, and ELISPOT assay provides a specific tool for early clinical laboratory diagnosis of paucibacillary (PB) leprosy cases; the methods are complementary to each other and beneficial for screening PB patients (Jiang et al., 2022).

Among these, quantitative PCR (qPCR) has emerged as a valuable tool due to its high sensitivity, specificity, and rapid turnaround time. qPCR not only amplifies the target DNA sequences but also allows for the quantification of the amount of *M. leprae* DNA present in the samples. Various clinical samples, including slit skin smears, skin biopsies, nasal swabs, and blood, have been explored for the detection of *M. leprae* DNA by targeting different genes (Martinez et al., 2014; Reis et al., 2014; Yan et al., 2014; Azevedo et al., 2017; Gama et al., 2018).

One of the most commonly used gene targets for qPCR in leprosy diagnosis is the *Rlep* gene (Gobbo et al., 2022; Braet et al., 2018; Carvalho et al., 2018; Martinez et al., 2011; Araujo et al., 2016; da Silva et al., 2021), 16srRNA (Martinez et al., 2010; Marques et al., 2018; Manta et al., 2019) and combined *Rlep*/16sRNA (Beissner et al., 2019). The *Rlep* gene is present in multiple copies within the *M. leprae* genome. The high copy number of the *Rlep* gene enables qPCR assays to achieve high sensitivity and specificity, even in paucibacillary cases (Barbieri et al., 2014; Barbieri et al., 2019). Skin biopsies are considered the gold standard for leprosy diagnosis as they provide direct access to the site of infection (Reibel et al., 2015; Tatipally et al., 2018). According to the studies mentioned above, the *Rlep* gene has been used in invasive samples such as SSS (Azevedo et al., 2017; Gobbo et al., 2022; da Silva et al., 2021), skin biopsies (Azevedo et al., 2017), and blood samples (Santos et al., 2001; Reis et al., 2014; Goulart et al., 2015; Araujo et al., 2016) and non-invasive samples such as nasal swabs (Araujo et al., 2016) and urine (David et al., 2024). The choice of sample type, gene target, and PCR technique employed can influence the effectiveness of the diagnosis.

Urine samples offer a non-invasive and easily accessible specimen for diagnostic testing, holding immense potential for identifying *M. leprae* DNA. The presence of *M. leprae* DNA in urine has been previously documented with varying degrees of sensitivity (Caleffi et al., 2012; David et al., 2024). This study presents a novel diagnostic strategy for leprosy patients by employing *Rlep* qPCR to detect *M. leprae* DNA in urine samples. The research involved a cohort of leprosy-affected individuals and non-leprosy control subjects. The aim of this work was to evaluate the sensitivity and specificity of qPCR and conventional PCR for the detection of *M. leprae* in urine samples among new untreated patients, in patients on MDT, and in patients who have been released from treatment.

## Materials and methods

### Sample collection and processing

A total of 101 subjects were enrolled in this study, including leprosy patients and healthy controls. Seventy-seven patients diagnosed with leprosy were selected, with 69 from a previous study and eight additional patients. Additionally, 24 non-leprosy cases (healthy individuals) were enrolled as controls.

Samples, including slit skin smears, blood, and urine, were collected. The slit skin smear test was performed for leprosy cases, and the bacteriological index (BI) was calculated according to the Ridley scale. Tissue material for PCR was collected either from the earlobe or forehead by slit skin scraping according to the instructions given by Turankar et al. (2014) and stored at −20°C. Blood and urine were obtained from leprosy patients, while only urine samples were taken from healthy individuals after obtaining informed consent for participation. The urine samples underwent routine analysis/microscopic tests, while the other samples were either processed promptly or immediately aliquoted and stored at temperatures of −20°C or −80°C for future use. DNA was extracted from blood, slit skin smear, and urine samples by using DNeasy Blood and Tissue Kit (Cat no: 69506) according to the manufacturer’s instructions. In brief, the slit skin smear samples were stored in 70% ethanol, thawed, and centrifuged at 10,000× rpm for 15 min, and the supernatant containing 70% ethanol was discarded. Then, DNA was extracted from the pellet according to protocols described by the manufacturer. For peripheral blood and urine samples, 200 µL of each was used, and DNA was extracted using the same protocol as described previously. The DNA was quantified using a NanoDrop 2000 spectrophotometer (Thermo Scientific), with ranges varying across sample types (David et al., 2024).

### Conventional PCR

PCR amplifications were carried out in an Agilent Surecycler 8,800 (Agilent Technologies) by using an *M. leprae*-specific *Rlep* gene target (129 bp) as described by Gama et al. (2018). A total of 20 µ l of reaction volume was prepared using 10 µL of 1X Hi-chrom PCR master mix (Cat. No: MBT089, HIMEDIA labs, Mumbai, India), 0.5 µL of 0.25 µM of each of forward (5′-TGCATGTCATGGCCTTGAGG-3′) and reverse (5′-CACCGATACCAGCGGCAGAA-3′) primers, 7 µL of nuclease-free water, and 2 µL of DNA. The reaction conditions included an initial denaturation of 95°C for 7 min, one cycle of 94°C for 2 min, annealing at 58°C for 2 min, and extension at 72°C for 2 min, followed by 40 cycles of 94°C for 30 s, 58°C for 30 s, and 72°C for 45 s. The reaction was terminated with a final extension of 72°C for 10 min, and the tubes were cooled to 25°C for 10 min. The amplicons were electrophoresed on a 2% agarose gel, and gel images were captured using Gel Doc™-XR+ Image Lab™ software (Cat No: 1708181). In this experiment, the DNA extracted from *M. leprae* strain Br4923 (Courtesy: BEI Resources, catalog number: NR-19351) was used as a positive control. The amplicons of urine samples were sent to a commercial facility (Symbiont Life Science, Chennai, India) for Sanger sequencing to confirm the sequence of the *Rlep* gene target (David et al., 2024).

### Quantitative PCR

qPCR was performed on a Rotor-Gene Q PCR system (S No.: R04114139; Qiagen Inc.), using *Rlep* primer (129 bp). A standard was prepared using the genomic DNA of *M. leprae* strain NHDP63 (Biodefense and Emerging Infections Research Resources Repository, BEI Resources) by serial dilution from 1 ng to 0.015 ng. qPCR was performed in triplicate for each dilution using 10 µL of QuantiTech SYBR Green PCR Master Mix (Cat.No.: 204143; Qiagen Inc.), 0.5 µL of 0.25 µM of each of forward and reverse primer for RLEP, and 7 µL of nuclease-free water. The reactions were cycled at 95°C for 10 min for one cycle, 94°C for 30 s, 58°C for 45 s, and 72°C for 45 s for 40 cycles, followed by melting curve analysis from 72°C to 95°C to determine nonspecific amplifications. The data were analyzed using Rotor-Gene Q series software. Data were acquired on the green channel during the annealing phase. In addition, qPCR was performed for all three clinical samples (SSS, blood, and urine) in duplicate using 2 µL of total DNA using the above cycling condition.

### Statistical analysis

A standard linear regression analysis of DNA from *M. leprae* vs. cycle threshold (Ct) values was calculated automatically by the Rotor-Gene Q series software. The data were transcribed into an MS Excel sheet, which was structured with data validation measures and then cross-verified by two independent researchers for accuracy. Following this, all statistical computations were performed using STATA version 16.0. The data were methodically presented through the utilization of frequency distributions, percentages, mean, median, and standard deviation values. The chi-square test was employed to establish the associations between leprosy classification and PCR outcomes (Ct value) from smear, blood, and urine samples. Similarly, contrasts were drawn among slit skin smear results, untreated new leprosy group, MDT group, and other pertinent groups. McNemar’s chi-square test was used to compare the PCR outcomes from various clinical samples across the study group. Disparities in Ct mean values between smear-positive and smear-negative results, as well as within categories such as pure neuritic leprosy (PNL), tuberculoid, and lepromatous forms across groups like untreated, MDT, and released from treatment (RFT) were analyzed using t-tests. The correlation between Ct values in diverse clinical samples from tuberculoid and lepromatous forms was evaluated through Pearson’s correlation analysis. A dot plot was employed to visually represent the comparative outcomes of qPCR and conventional PCR across various clinical samples, as well as in relation to BI grading and qPCR results and throughout RJ classification. A significance level of p < 0.05 was deemed as statistically significant for all analyses.

## Results

In an attempt to identify a sensitive and precise technique for detecting *M. leprae* DNA, qPCR was performed on different clinical samples using the *Rlep* gene target. Furthermore, the qPCR results were compared to the conventional PCR results from a previous study (David et al., 2024). The sensitivity of the qPCR was evaluated for the *Rlep* gene target by utilizing standard DNA from the *M. leprae* strain NHDP63. The critical threshold fluorescence values were recorded for standard DNA dilutions with a negative cutoff value of >38 and are represented in Table 1.

**TABLE 1:** Normalized CT values for the *Rlep* gene target (*M.leprae* strain NHDP63).

*M.leprae* genomic DNA dilutions (ng)	CT value	Copy number
1	15.56	4.086× 10^8^
0.5	17.25	2.042× 10^8^
0.25	18.67	1.021× 10^8^
0.125	20.33	5.108× 10^7^
0.0625	21.51	2.553 × 10^7^
0.03	24.51	1.225× 10^7^
0.015	34.17	6.129× 10^6^

In this study, 101 subjects were included; 77 (76.24%) were leprosy patients, and 24 (23.76%) were healthy individuals. Of 101, 51 (50.5%) were men and 50 (49.5%) were women, and patients were diagnosed with leprosy by clinical and bacilloscopy analysis. Among 77 leprosy patients, 33 were new untreated patients, 27 were on MDT, and 17 patients had been released from treatment. Fifty of these patients were smear-negative, and 27 were smear-positive. The demographics, clinical classifications, and BIs for both the leprosy and control groups are described in Table 2.

**TABLE 2:** Clinical, demographic, classification, and bacteriological index data on leprosy and non-leprosy among the study group.

S.no	Characteristics	Types	*NEW LEPROSY (n = 33)		ON MDT (n = 27)		RFT (n = 17)		CONTROL (n = 24)		TOTAL (n = 101)	
			No	%	No	%	No	%	No	%	No	%
1	Gender	Male	20	60.6	13	48.1	9	52.9	09	62.5	51	50.5
		Female	13	39.4	14	51.9	8	47.1	15	37.5	50	49.5
2	Age	<15	01	3.0	02	7.4	-	-	01	4.2	04	4.0
		16–30	07	21.2	08	29.6	03	17.6	07	29.2	25	24.8
		31–50	18	54.5	10	37.0	8	47.1	06	25.0	42	41.5
		Above 51	07	21.2	07	26.0	6	35.3	10	41.6	30	29.7
3	RJ	PNL	06	18.2	02	7.4	-	-	-	-	08	10.3
		TT	-	-	01	3.7	-	-	-	-	01	1.3
		BT	15	45.5	04	14.8	06	35.2	-	-	25	32.5
		BB	02	6.1	01	3.7	-		-	-	03	3.9
		BL	05	15.1	07	26.0	06	35.2	-	-	18	23.4
		LL	05	15.1	12	44.4	05	29.4	-	-	22	28.6
4	BI	0	24	72.7	13	48.2	13	76.5	-	-	50	64.9
		1–4	09	27.3	14	51.8	04	23.5	-	-	27	35.1

Of 77 leprosy patients, eight were pure neuritic leprosy (PNL), 25 were borderline tuberculoid (BT), one was tuberculoid tuberculoid (TT), three were borderline borderline (BB), 18 were borderline lepromatous (BL), and 22 were lepromatous leprosy (LL) (Table 2).

The qPCR results for the detection of *M. leprae* DNA in various clinical samples (smear, blood, and urine) from both leprosy patients and the non-leprosy group are shown in Table 3.

**TABLE 3:** qPCR results according to the leprosy classification and the type of samples.

Clinical form/type of sample	qPCR results		Ct value				p-value
	Positive	Negative	Range	Mean	P50	SD	
qPCR of smear sample							
1. PNL (n = 8)	07 (87.5%)	01 (12.5%)	27.93–32.48	32.25	31.9	1.75	0.510
2. Tuberculoid (n = 26)	23 (88.5%)	03 (11.5%)	29.51–36.06	30.31	30.13	1.81	
3. Lepromatous (n = 43)	41 (95.3%)	02 (4.7%)	**26.29**–35.66	30.58	30.3	2.35	
qPCR of blood sample							
1. PNL (n = 8)	08 (100%)	-	27.07–34.99	29.45	29.35	1.8	0.447
2. Tuberculoid (n = 26)	24 (92.3%)	02 (8.7%)	26.77–33.89	30.21	29.70	2.9	
3. Lepromatous (n = 43)	42 (97.7%)	01 (2.3%)	**25.16**–33.87	29.01	28.96	2.00	
qPCR of urine sample							
1. PNL (n = 8)	08 (100%)	-	27.25–33.87	31.48	31.9	2.2	-
2. Tuberculoid (n = 26)	26 (100%)	-	**25.49**–35.93	29.59	29.39	2.04	
3. Lepromatous (n = 43)	43 (100%)	-	26.14–35.1	30.86	30.46	2.04	
4. Control (n = 24)	-	24 (100%)	-	-	-	-	

Seven of eight PNL patients showed PCR positivity in smear samples (87.5%), while all eight were positive in both urine and blood samples (100%). The Ct values ranged from 27.93 to 32.48 (mean of 32.25 ± 1.75) in the smear, from 27.07 to 34.99 (mean of 29.45 ± 1.8) in blood, and from 27.25 to 33.87 (mean of 31.48 ± 2.2) in urine (Table 3).

Among 26 patients in the tuberculoid pole, 23 showed PCR positivity in smear samples (88.5%), 24 showed positive in blood samples (92.3%), and all 26 were positive in urine samples (100%). The Ct values ranged from 29.51 to 36.06 (mean of 30.31 ± 1.81) in smear, from 26.77 to 33.89 (mean of 30.21 ± 2.9) in blood, and from 25.49 to 35.93 (mean of 29.59 ± 2.04) in urine (Table 3).

Of 43 patients in the lepromatous pole, 41 showed PCR positivity in smear samples (95.3%), 42 showed a positive result in blood samples (97.7%), and all 43 showed a positive result in urine samples (100%). The Ct values ranged from 26.29 to 35.66 (mean of 30.58 ± 2.35) in smear, from 25.16 to 33.87 (mean of 29.01 ± 2.00) in blood, and from 26.14 to 35.1 (mean of 30.86 ± 2.06) in urine. All 24 controls showed negative PCR in urine samples (Table 3).

These results indicated the highest PCR positivity in various clinical samples, including smears, blood, and urine samples.

All three clinical forms (PNL, tuberculoid, and lepromatous) exhibited 100% PCR positivity in urine samples, with tuberculoid showing the lowest Ct value, ranging from 25.49 to 35.93. Differences in bacterial load and disease severity are reflected in the variation of Ct values between various sample types and leprosy classifications.

The qPCR results in Table 4 display the detection of *M. leprae* DNA in various clinical samples (skin smears, blood, and urine) categorized according to the bacterial index (BI) grading obtained from slit skin smear examinations.

**TABLE 4:** PCR results according to slit skin smear (BI) results among all the study groups.

BI grading/type of sample	qPCR results		Ct value				p-value
	Positive	Negative	Range	Mean	P50	SD	
1. qPCR of the smear sample							
BI negative (n = 50)	45 (90%)	05 (10%)	27.93–36.06	31.91	31.89	1.9	0.325
BI positive (n = 27)	26 (96.3%)	01 (3.7%)	26.29–33.28	29.69	29.36	2.04	
2. qPCR of the blood sample							
BI negative (n = 50)	47 (94%)	03 (06%)	25.16–34.99	29.39	29.3	2.1	0.194
BI positive (n = 27)	27 (100%)	-	25.27–33.87	29.10	28.96	1.95	
3.qPCR of the urine sample							
BI negative (n = 50)	50 (100%)	-	25.49–35.93	30.91	30.78	2.21	-
BI positive (n = 27)	27 (100%)	-	26.43–35.1	31.00	30.46	2.11	

qPCR results also indicated the presence of *M. leprae* DNA targeting the *Rlep* gene (129 bp) both in patients with negative skin smears and in patients with positive skin smears. Smear-negative patients (BI negative) had a high rate of PCR positivity in urine samples, followed by blood samples at 94% and smear samples at 90%. Smear-positive patients (BI positive) had a PCR positivity rate of 100% in both urine and blood samples, followed by a rate of 96.3% in smear samples (Table 4).

In urine samples, the Ct values ranged from 25.49–35.93 (mean of 30.91 ± 2.21) in smear-negative patients and 26.43–35.1 (mean of 31.00 ± 2.11) in smear-positive patients. Conversely, the blood samples had low Ct values ranging from 25.16–34.99 (mean of 29.39 ± 2.1) in smear-negative and 25.27–33.87 (mean of 29.10 ± 1.95) in smear-positive patients. In contrast, smear samples showed Ct value ranging from 27.93–36.06 (mean of 31.91 ± 1.9) in smear-negative patients and 26.29–33.28 (29.69 ± 2.04) in smear-positive patients (Table 4).

Among new untreated patients who were smear-negative, the highest percentage of PCR positivity was observed in urine samples (100%), followed by blood (95.8%) and skin smears (91.7%). In contrast, all three samples showed a PCR positivity rate of 100% in smear-positive patients. The smear-negative patients had low Ct values in urine samples, followed by blood and smear samples. The smear-positive patients had low Ct values in smear samples, followed by blood and urine samples, respectively (Table 5).

**TABLE 5:** qPCR results according to classification and clinical samples in the new untreated leprosy group (n = 33).

BI grading/clinical form	Clinical	qPCR results		Ct value				p-value
	Samples	Positive	Negative	Range	Mean	P50	SD	
BI negative (n = 24)	1. Smear 2. Blood 3. Urine	22 (91.7%) 23 (95.8%) 24 (100%)	02 (8.3%) 01 (4.2%) -	27.93–36.06 25.59–34.99 25.49–34.44	31.84 29.40 30.04	31.77 28.87 29.97	1.96 2.26 1.90	0.006 0.003 0.062
BI positive (n = 09)	1. Smear 2. Blood 3. Urine	09 (100%) 09 (100%) 09 (100%)	- - -	26.29–32.16 27.41–31.91 30.21–35.1	29.06 29.64 32.22	29.24 29.72 32.63	2.05 1.53 1.52	1.000 1.000 1.000
PNL (n = 06)	1. Smear 2. Blood 3. Urine	06 (100%) 06 (100%) 06 (100%)	- - -	27.93–32.48 27.07–34.99 27.57–30.53	30.65 30.63 29.27	30.62 29.74 29.39	1.72 3.25 1.00	0.500 0.500 1.000
Tuberculoid (n = 15)	1. Smear 2. Blood 3. Urine	13 (86.7%) 14 (93.3%) 15 (100%)	02 (13.3%) 01 (6.7%) -	29.51–36.06 26.77–32.5 25.49–34.44	32.32 29.23 30.45	31.98 29.1 30.45	1.84 1.57 2.25	0.039 0.062 0.062
Lepromatous (n = 12)	1. Smear 2. Blood 3. Urine	12 (100%) 12 (100%) 12 (100%)	- - -	26.29–34.59 25.59–31.91 28.77–35.1	29.82 29.17 31.56	29.41 29.67 31.57	2.49 1.84 1.79	0.500 0.031 0.004

In the new untreated group, patients with no skin lesions categorized as PNL demonstrated the highest PCR positivity at 100% in all three samples: smears, blood, and urine. However, urine samples showed low Ct values between 27.57 and 30.53 (average of 29.27 ± 1.00) (Table 5). Among tuberculoid pole patients, PCR positivity was observed at 100% in urine samples, followed by 93.3% in blood and 86.7% in smear samples. Urine samples had anticipated low Ct values, followed by smear samples. In the lepromatous group, all three samples showed PCR positivity at 100%. As expected, low Ct values were found in blood and smear samples, followed by urine samples (Table 5).

The Patients with negative smears in the MDT group, PCR positivity was highest in urine samples (100%), followed by 92.3% in blood and 84.6% in smear samples. For patients under MDT who were smear-positive, PCR positivity was at 100% in blood and urine samples and 92.9% in smear samples. The smear-negative individuals showed lower Ct values in blood, followed by urine and smears, respectively. The smear-positive patients had low Ct values in blood, followed by smear and urine samples, respectively (Table 6).

**TABLE 6:** qPCR results according to classification and clinical samples in the MDT group (n = 27).

BI grading/clinical form	Clinical	qPCR results		Ct value				p-value
	Samples	Positive	Negative	Range	Mean	P50	SD	
BI negative (n = 13)	1. Smear 2. Blood 3. Urine	11 (84.6%) 12 (92.3%) 13 (100%)	02 (15.4%) 01 -	28.25–33.12 25.16–32.31 27.25–33.87	30.94 29.00 31.56	31.07 29.09 32.38	1.38 2.13 1.98	0.015 0.031 0.003
BI positive (n = 14)	1. Smear 2. Blood 3. Urine	13 (92.9%) 14 (100%) 14 (100%)	01 (7.1%) - -	26.62–32.85 25.27–33.87 28.79–34.35	29.62 28.82 30.88	29.32 28.61 30.3	1.91 2.23 1.81	1.000 1.000 1.000
PNL (n = 02)	1. Smear 2. Blood 3. Urine	01 (50%) 02 (100%) 02 (100%)	01 (50%) - -	28.25 27.36–30.59 27.25–33.87	28.25 28.97 30.56	28.25 28.97 30.56	- 2.28 4.68	1.000 1.000 1.000
Tuberculoid (n = 05)	1. Smear 2. Blood 3. Urine	05 (100%) 05 (100%) 05 (100%)	- - -	30.11–32.3 27.74–32.31 30.9–33.48	31.54 29.69 32.47	31.57 29.58 32.72	0.87 1.97 0.97	0.062 0.250 0.062
Lepromatous (Yan et al., 2014)	1. Smear 2. Blood 3. Urine	18 (90%) 19 (95%) 20 (100%)	02 (10%) 01 (5%) -	26.62–33.12 25.16–33.87 28.79–34.35	29.97 28.69 30.96	29.74 28.87 30.3	1.82 2.24 1.73	0.317 0.003 0.007

In the same MDT group, tuberculoid patients showed high PCR positivity at 100% in all three clinical samples. As expected, tuberculoid had a high Ct value in urine samples, followed by smear and blood samples, respectively. The lepromatous patients demonstrated PCR positivity in all three samples, with 90% in smear, 95% in blood, and 100% in urine samples. Lepromatous patients displayed low Ct values in blood, with similar values observed in smear and urine samples, respectively (Table 6).

Among the RFT group, smear-negative and smear-positive patients showed the highest PCR positivity at 100% in urine samples. The tuberculoid patients in the RFT group showed PCR positivity in all three samples at 83.3% in smear and blood samples and in urine at 100%. Meanwhile, the lepromatous group showed PCR positivity at 100% in all three samples. As expected, lepromatous patients had low Ct values in urine samples, followed by blood and smear samples. Conversely, tuberculoid patients had high Ct values in urine samples, followed by smear and blood samples (Table 7).

**TABLE 7:** qPCR results according to classification and clinical samples in the RFT group (n = 17).

BI grading/clinical form	Clinical	qPCR results		Ct value				p-value
	Samples	Positive	Negative	Range	Mean	P50	SD	
BI negative (n = 13)	1. Smear 2. Blood 3. Urine	12(92.3%) 12(92.3%) 13(100%)	01(7.7%) 01(7.7%) -	30.18–36.03 27.51–33.89 26.14–35.93	32.94 29.78 31.85	32.63 29.30 32.43	2.04 2.01 2.50	0.002 0.002 0.002
BI positive (n = 04)	1. Smear 2. Blood 3. Urine	04(100%) 04(100%) 04(100%)	- - -	29.21–33.28 27.4–31.67 26.43–31.16	31.35 28.85 28.65	31.46 28.16 28.52	2.01 1.92 2.50	1.000 1.000 1.000
Tuberculoid (n = 06)	1. Smear 2. Blood 3. Urine	05(83.3%) 05(83.3%) 06(100%)	01(16.7%) 01(16.7%) -	30.18–36.03 27.51–33.89 30.98–35.93	32.79 29.85 33.23	31.98 29.38 32.89	2.23 2.50 1.74	0.062 0.125 0.031
Lepromatous (n = 11)	1. Smear 2. Blood 3. Urine	11 (100%) 11 (100%) 11 (100%)	- - -	29.21–35.66 27.4–33.03 26.14–33.09	32.42 29.41 29.93	32.85 28.97 30.46	2.12 1.80 2.60	0.015 0.015 0.062

Of eight PNL patients, six people from the untreated group tested positive (100%) for PCR in all three samples, with urine samples showing low Ct values ranging from 27.57–30.53 (average of 29.27 ± 1.00). Blood and smear samples had Ct values ranging from 27.07–34.99 (mean of 30.63 ± 3.25) and 27.93–32.48 (mean of 30.65 ± 1.72), respectively. In the MDT group, two PNL patients tested positive for PCR in both blood and urine samples, while smear samples showed 50% positivity with a Ct value of 28.25 (Table 8).

**TABLE 8:** qPCR results according to the Ridley–Jopling classification and clinical samples among groups (n = 77).

RJ classification	New untreated (n = 33) Ct value ranges			On MDT (n = 27) Ct value ranges			RFT (n = 17) Ct value ranges		
	Smear	Blood	Urine	Smear	Blood	Urine	Smear	Blood	Urine
PNL	27.93–32.48 (100%)	27.07–34.99 (100%)	27.57–30.53 (100%)	28.25 (50%)	27.36–30.59 (100%)	27.25–33.87 (100%)	-	-	-
TT	-	-	-	30.11	32.31	32.3	-	-	-
BT	29.51–36.06 (86.6%)	26.77–32.5 (93.3%)	25.49–34.44 (100%)	31.53–32.3 (100%)	27.74–30.96 (100%)	30.9–33.48 (100%)	30.18–36.03 (83.3%)	27.51–33.89 (83.3%)	30.98–35.93 (100%)
BB	29.24–34.59 (100%)	29.77–30.13 (100%)	29.67–35.1 (100%)	30.01	25.16	29.16	-	-	-
BL	29.4–32.33 (100%)	25.59–31.91 (100%)	28.77–32.67 (100%)	29.88–33.12 (71.4%)	26.72–31.59 (85.7%)	29.87–33.09 (100%)	30.07–35.66 (100%)	27.52–31.15 (100%)	30.29–33.09 (100%)
LL	26.29–30.49 (100%)	27.41–29.77 (100%)	30.25–33.01 (100%)	26.62–32.85 (100%)	25.27–33.87 (100%)	28.79–34.35 (100%)	29.21–35.65 (100%)	27.4–33.03 (100%)	26.14–30.46 (100%)

### Statistical results

Comparative analysis between the slit skin smear test and qPCR was conducted for different clinical samples across all study groups. In the untreated group, statistically significant differences (p-value <0.05) were observed in the smear and blood samples between BI-negative and BI-positive individuals, as well as in the blood and urine samples of lepromatous cases (Table 5). In the treated group, statistically significant differences were found in the Ct values of lepromatous patients’ blood and urine samples, indicating higher detection sensitivity in these samples (Table 6). In the RFT group, significant p-values (0.002) for BI-negative individuals in all sample types suggest notable differences in Ct values compared to BI-positive individuals (Table 7).

**FIGURE 1:**
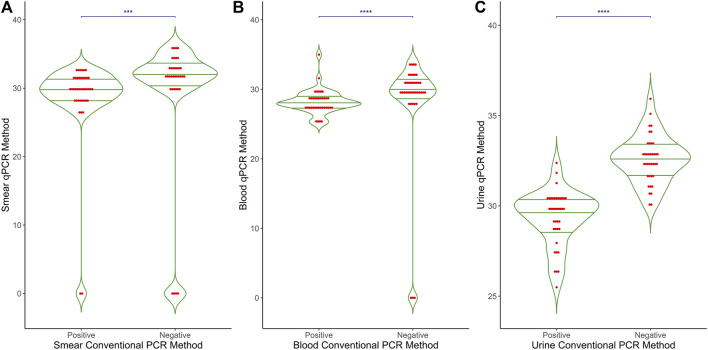
Comparative results of qPCR and conventional PCR in different clinical samples. The graph presents a comparison between the results of Conventional PCR (X-axis) and qPCR (Y-axis) across three different clinical sample types: **(A)** Smear **(B)** Blood and **(C)** Urine. The X-axis represents the outcomes of Conventional PCR, while the Y-axis displays the Ct values obtained from qPCR. This graphical representation illustrates the relationship and differences between the two PCR methods in detecting target DNA in the samples.

Figure 1 shows a comparative analysis of the Ct values obtained from qPCR methods in relation to the conventional PCR method results (positive vs. negative) across three different types of samples: (A) smear, (B) blood, and (C) urine. Each plot displays the distribution of Ct values for the qPCR method, with individual data points represented as red dots and mean values indicated by a horizontal green line. The p-values for the differences between positive and negative groups in all sample types are <0.0001, indicating a highly significant difference between the qPCR Ct values of conventional-PCR-positive samples and those that are conventional-PCR-negative. Negative results from conventional PCR consistently show no amplification in the qPCR method, reinforcing the reliability and specificity of the qPCR approach in detecting *M. leprae* DNA.

**FIGURE 2:**
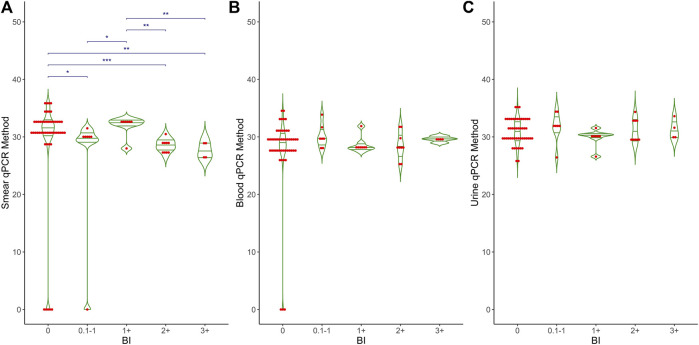
Comparative analysis of BI grading and qPCR results in different clinical samples. This graph shows the relationship between BI grading (X-axis) and qPCR Ct values (Y-axis) across three different clinical samples: **(A)** Smear, **(B)** Blood and **(C)** Urine. The X-axis represents Bacteriological Index (BI) grading, ranging from 0 to 3+, while the Y-axis shows the Ct values obtained from qPCR. The graph also includes mean, median, and *p*-values to highlight the statistical analysis and differences observed in the qPCR results relative to BI grading.

Figure 2 shows the Ct values obtained from the qPCR method in relation to the bacterial index (BI) for three types of samples: (A) smear, (B) blood, and (C) urine. The BI grading is displayed along the *x*-axis, ranging from 0 (no bacteria detected) to 3+ (high bacterial load), with intermediate categories (0.1–1+, 0.2+, 0.3+, 1+, 1+<2+, 1+<3+). Each plot includes individual data points (red dots) and mean values (horizontal green lines) for the qPCR Ct values. For smear samples, there is a significant difference (p < 0.001) in Ct values across different BI categories, while no significant differences were observed for blood (p = 0.456) and urine samples (p = 0.594). Overall, Ct values are approximately 30 across all BI categories for all sample types, with some variability within each category. A thin horizontal blue line along with the asterisks (^*^p < 0.05, ^**^p < 0.01, and ^***^p < 0.001) indicates the significant difference between BI grading and smear-qPCR. No significant differences were observed for blood and urine samples.

**FIGURE 3:**
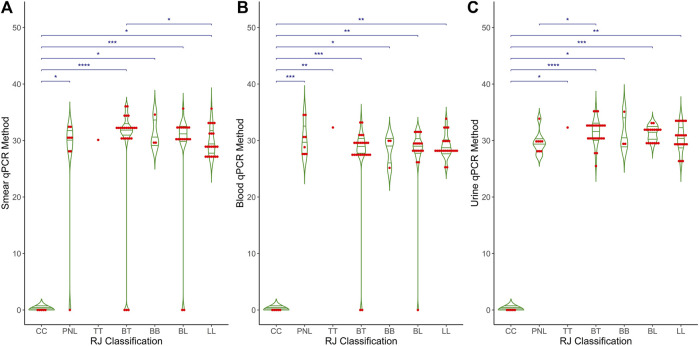
Comparative analysis of qPCR results across RJ classification in different clinical samples. The graph compares RJ classification (X-axis) with qPCR Ct values (Y-axis) across three distinct clinical samples: **(A)** Smear, **(B)** Blood and **(C)** Urine. The X-axis represents the RJ classification categories, while the Y-axis displays the Ct values obtained from qPCR. The graph also includes statistical measures, such as mean, median, and *p*-values, to highlight differences and trends in qPCR results relative to RJ classification.

Figure 3 shows the distribution of Ct values obtained from the qPCR method across different clinical forms of leprosy and control groups for three types of samples: (A) smear, (B) blood, and (C) urine. Each plot includes individual data points (red dots) and mean values (horizontal green lines) for the qPCR Ct values. Control cases show no detectable Ct values, indicating no amplification. In contrast, clinical forms of leprosy exhibit a range of Ct values from 25 to 40, with mean Ct values around 30. Smear samples showed significant differences (p < 0.001) between the control and the different clinical forms and between BT and LL. Both blood and urine samples showed significant differences (p < 0.001) between the control and the different clinical forms and between BT and PNL. These results demonstrate the variability in bacterial load and detection sensitivity across different sample types and clinical manifestations of leprosy. A thin horizontal blue line along with the asterisks (^*^p < 0.05, ^**^p < 0.01, and ^***^p < 0.001) indicates the significant difference within clinical groups, such as control vs. PNL, control vs. tuberculoid, and control vs. lepromatous in different clinical samples.

**FIGURE 4:**
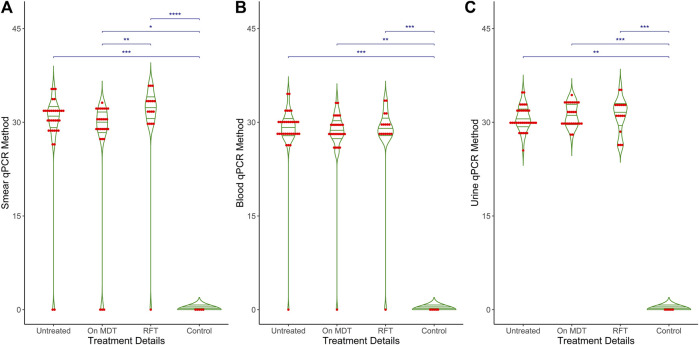
Comparative analysis of qPCR results among study groups in different clinical samples. The graph depicts the relationship between different treatment groups (X-axis: Untreated, MDT, and RFT) and qPCR Ct values (Y-axis) across three distinct clinical samples: **(A)** Smear, **(B)** Blood and **(C)** Urine. The X-axis represents the treatment groups, while the Y-axis shows the Ct values obtained from qPCR. Additionally, the graph includes mean, median, and p-values to illustrate the statistical differences in qPCR results among the treatment groups.

Figure 4 shows the Ct values obtained from the qPCR method across different study groups of leprosy and control cases for three types of samples: (A) smear, (B) blood, and (C) urine. Each plot includes individual data points (red dots) and mean values (horizontal green lines) for the qPCR Ct values. Control cases consistently show no detectable Ct values, indicating no amplification. In contrast, clinical forms of leprosy exhibit a range of Ct values from 25 to 40, with mean values around 30. There are significant differences in Ct values between control cases and clinical categories (p < 0.001), as well as specific differences within clinical categories (p < 0.001), reflecting the variability in bacterial load and detection sensitivity across different sample types and clinical manifestations of leprosy. A thin horizontal blue line along with the asterisks (^*^p < 0.05, ^**^p < 0.01, and ^***^p < 0.001) indicates the significant difference within clinical groups, such as control vs. untreated, control vs. MDT, and control vs. RFTs in different clinical samples.

## Discussion

This study presents the first systematic comparative evaluation of conventional and quantitative PCR for detecting *M. leprae* DNA in urine samples from leprosy patients with different clinical forms of the disease, as well as from healthy individuals. Our previous work demonstrated that the *Rlep* gene could be detected in urine samples of leprosy patients using the conventional PCR method, with no amplification observed in urine samples of non-leprosy patients (David et al., 2024). No consistent amplification was observed in any of the urine samples taken from healthy individuals.

Several studies have been published on PCR-based methods to detect *M. leprae* DNA in various clinical samples using different gene targets. Truman et al. (2008) quantified *M. leprae* DNA in mice footpad (MFP) models, showing greater sensitivity and reproducibility than direct microscopic examination (Truman et al., 2008). Martinez et al. reported an 87.1% sensitivity for the *Rlep* gene in skin biopsies using qPCR, demonstrating its efficacy over other gene targets such as sodA, 16S rRNA, and Ag 85B (Martinez et al., 2011).

Goulart et al. showed that three gene targets (Rlep, 16s rRNA, and Ag 85B) were the most sensitive and specific for detecting *M. leprae* DNA among those genes discussed in the literature (Goulart and Goulart, 2008). Mohanty et al. recently validated that the *Rlep* gene demonstrated superior specificity and sensitivity compared to other genes when using conventional PCR (Mohanty et al., 2020).

Previous studies have reported the detection of *M. leprae* DNA using real-time PCR in both invasive samples (slit skin smear samples, blood, and biopsies) and non-invasive samples (nasal swabs, nasal secretion, and urine samples) with different gene targets. Azevedo et al. showed PCR positivity at 84% in smear samples and 84.9% in skin biopsies using the *Rlep* gene by qPCR (Azevedo et al., 2017). Da Silva et al. showed PCR positivity at 83.9%, and Gobbo et al. showed 86.07% in smear samples of new leprosy cases using *Rlep* qPCR (Gobbo et al., 2022; da Silva et al., 2021). Araujo et al. demonstrated a PCR positivity rate of 66.4% in nasal swabs by targeting the *Rlep* gene (Araujo et al., 2016).

Other types of clinical samples were also investigated for the detection of *M. leprae* DNA. Reis et al. performed qPCR on peripheral blood using an ML0024 target and showed a 22% PCR positivity rate (Reis et al., 2014), and Turankar et al. performed conventional PCR on blood and showed 100% PCR positivity in blood samples of leprosy patients (Turankar et al., 2015). However, no additional studies are available regarding the use of the *Rlep* gene target for qPCR detection of *M. leprae* DNA in blood samples.

Three studies have reported the detection of *M. leprae* DNA in urine samples of leprosy patients. Parkash et al. and Caleffi et al. showed the first and second results using the Pra gene with varied fragment lengths, exhibiting PCR positivity rates of 37.5% (6/16) and 46.6% (34/73) (Parkash et al., 2004; Caleffi et al., 2012). The third was our study, David et al., which used the *Rlep* gene target and showed the detection of *M. leprae* DNA in urine samples of leprosy patients (David et al., 2024). All three studies demonstrated the presence *of M. leprae* DNA in urine samples and detected varying levels of PCR positivity without quantifying the amount of bacilli.

The current study demonstrated that *M. leprae* DNA could be quantified in different clinical samples, such as slit skin smears, blood, and urine samples, using the *Rlep* gene target and qPCR across different clinical forms of leprosy among different study groups, including new untreated, MDT, and RFT. Urine and blood samples showed higher PCR positivity than smear samples among all the study groups.

The results among new untreated leprosy patients showed that PCR positivity in urine samples was 100% in smear-negative tuberculoid patients, with Ct values ranging from 25.49–34.44. Similarly, it is interesting that smear-positive lepromatous patients showed 100% in urine samples, with Ct values ranging from 28.77–35.1. Blood samples showed low Ct values (25.59–32.5) compared to smear samples (26.29–36.06) across tuberculoid and lepromatous pole patients. Thus, there seems to be an inverse relationship between smear positivity and severe forms of leprosy and the presence of PCR positivity in urine samples. Additionally, detecting *M. leprae* DNA in urine samples can be used to confirm the diagnosis of PNL in the absence of skin lesions.

Our previous study (David et al., 2024) indicated that conventional PCR showed lower *M. leprae* DNA excretion in urine samples from smear-positive patients (high bacillary load) than smear-negative patients (low bacillary load). This study corroborates those findings with qPCR, demonstrating low Ct values in smear-negative patients and high Ct values in smear-positive patients.

Among treated patients who were skin smear-negative, PCR positivity was observed in all three clinical samples at varying levels. As expected, smear-positive patients showed higher PCR positivity levels in all three clinical samples. The differences in Ct values were also noted between tuberculoid and lepromatous pole patients, with tuberculoid patients showing high Ct values in urine samples and lepromatous patients showing low Ct values.

In this study, results among RFT patients showed high Ct values in smear samples of both the tuberculoid and lepromatous groups. Conversely, urine samples had low Ct values in both groups, particularly in the lepromatous groups, ranging from 26.14–33.09. This result suggests that serial PCR determination using urine samples could be beneficial for monitoring the effectiveness of MDT in both tuberculoid and lepromatous pole patients.

Overall, blood samples also showed promising results, with both qPCR and conventional PCR high levels of PCR positivity and low Ct values in lepromatous pole patients before and after treatment. Blood is associated with the persistence of dead *M. leprae* and the continuous elimination of its DNA fragments in urine (Santos et al., 2001; Caleffi et al., 2012). Further investigation is needed to determine whether quantifying *M. leprae* DNA in urine samples during and after MDT can help predict relapse or drug resistance and determine if the disease is cured.

As qPCR with the *Rlep* gene in urine samples has proven effective for detecting *M. leprae* in untreated smear-negative and treated smear-positive leprosy patients, it is important to investigate whether this method can also detect and measure the presence of *M. leprae* DNA in urine samples of leprosy contacts to identify infection among them. This research offers important information for the diagnosis and monitoring of leprosy treatment.

## Conclusion

This study provides comprehensive insights into the detection of *M. leprae* DNA in smear, blood, and urine samples of leprosy patients using qPCR and conventional PCR targeting the *Rlep* gene. Our findings suggest that the *Rlep* gene target is promising for PCR-based diagnosis of leprosy, particularly in urine samples from smear-negative tuberculoid and PNL patients. Additionally, this method can track the efficacy of MDT and other anti-leprosy treatments across different leprosy disease groups. Further studies in this area are required with a larger number of patients and monitoring of *M. leprae* DNA levels in urine in new untreated patients, from diagnosis to treatment monitoring to prediction of events such as relapse and drug resistance.

Despite the benefits of increased sensitivity and faster results, qPCR may not be feasible in all settings due to its expensive equipment, techniques, and reagents. Traditional PCR, although less sensitive, is still a useful tool, particularly in reference centers with limited resources or infrastructure.

The study also noted minimal fluctuation in the Ct values in urine samples from tuberculoid individuals, suggesting that qPCR is a reliable method for detecting *M. leprae* DNA load in this specific group of patients. In general, the potential of utilizing qPCR for diagnosing leprosy and tracking treatment progress is very promising. Expanding the application of qPCR outside of reference centers may decrease the chance of false-negative outcomes, rendering it a beneficial supplementary diagnostic tool in wider healthcare facilities.

## Data Availability

The raw data supporting the conclusions of this article will be made available by the authors, without undue reservation.
